# Correlation and Interaction Visualization of Altmetric Indicators Extracted From Scholarly Social Network Activities: Dimensions and Structure

**DOI:** 10.2196/jmir.2707

**Published:** 2013-11-25

**Authors:** Chun Li Liu, Yue Quan Xu, Hui Wu, Si Si Chen, Ji Jun Guo

**Affiliations:** ^1^Information OfficeLibraryChina Medical UniversityShenyang, LiaoningChina; ^2^Information Management DepartmentSchool of Computer Science and Information TechnologyNortheast Normal UniversityChangchun, JilinChina; ^3^Department of Social MedicineSchool of Public HealthChina Medical UniversityShenyang, LiaoningChina; ^4^Sector of Library DirectorLibraryChina Medical UniversityShenyang, LiaoningChina

**Keywords:** altmetrics, article-level metrics, scholarly social network tools, indicator, dimension, structure

## Abstract

**Background:**

Citation counts for peer-reviewed articles and the impact factor of journals have long been indicators of article importance or quality. In the Web 2.0 era, growing numbers of scholars are using scholarly social network tools to communicate scientific ideas with colleagues, thereby making traditional indicators less sufficient, immediate, and comprehensive. In these new situations, the altmetric indicators offer alternative measures that reflect the multidimensional nature of scholarly impact in an immediate, open, and individualized way. In this direction of research, some studies have demonstrated the correlation between altmetrics and traditional metrics with different samples. However, up to now, there has been relatively little research done on the dimension and interaction structure of altmetrics.

**Objective:**

Our goal was to reveal the number of dimensions that altmetric indicators should be divided into and the structure in which altmetric indicators interact with each other.

**Methods:**

Because an article-level metrics dataset is collected from scholarly social media and open access platforms, it is one of the most robust samples available to study altmetric indicators. Therefore, we downloaded a large dataset containing activity data in 20 types of metrics present in 33,128 academic articles from the application programming interface website. First, we analyzed the correlation among altmetric indicators using Spearman rank correlation. Second, we visualized the multiple correlation coefficient matrixes with graduated colors. Third, inputting the correlation matrix, we drew an MDS diagram to demonstrate the dimension for altmetric indicators. For correlation structure, we used a social network map to represent the social relationships and the strength of relations.

**Results:**

We found that the distribution of altmetric indicators is significantly non-normal and positively skewed. The distribution of downloads and page views follows the Pareto law. Moreover, we found that the Spearman coefficients from 91.58% of the pairs of variables indicate statistical significance at the .01 level. The non-metric MDS map divided the 20 altmetric indicators into three clusters: traditional metrics, active altmetrics, and inactive altmetrics. The social network diagram showed two subgroups that are tied to each other but not to other groups, thus indicating an intersection between altmetrics and traditional metric indicators.

**Conclusions:**

Altmetrics complement, and most correlate significantly with, traditional measures. Therefore, in future evaluations of the social impact of articles, we should consider not only traditional metrics but also active altmetrics. There may also be a transfer phenomenon for the social impact of academic articles. The impact transfer path has transfer, or intermediate, stations that transport and accelerate article social impact from active altmetrics to traditional metrics and vice versa. This discovery will be helpful to explain the impact transfer mechanism of articles in the Web 2.0 era. Hence, altmetrics are in fact superior to traditional filters for assessing scholarly impact in multiple dimensions and in terms of social structure.

## Introduction

The evaluation of an academic paper’s influence is important for scientists and academic management mechanisms [1]. Scientific papers are regarded by the scientific community as the formal carriers of recent findings and innovative ideas from scientific experiments [2]. Official periodicals are considered the major medium used in scientific communication [3]. Citation rates per paper and the impact factor per scientific journal have been used as evaluation indicators for measuring academic impact [4,5].

Impact factor is based on journals, not journal articles. It is unlikely that one type of metric (for example, citation counts) can adequately inform evaluations across multiple disciplines, departments, career stages, and job types. In addition, a newly published article requires time to accumulate citations—a citation delay may range from 3 months to 1-2 years, sometimes longer in formal publications. By contrast, only a few days are required to tabulate statistics from viewing, downloading, tags, digs, tweets, and blogs in scientific social networks.

A reasonable evaluation should include not only quantitative assessments but also the peer-review process. The traditional peer-review process has been criticized for its scalability, that is, the inability to cope with an increasingly large number of scientific paper submissions, given the limited number of available reviewers and publication time constraints.

With the development of the open access platform [6,7] and the practical application of academic social networks [8,9], scientific achievements have now been able to spread more rapidly [10-13]. Given these new developments, the open access platforms, social network tools, and other online usage and comment-based statistics have been paving the way to new forms of scientific evaluation, which could complement traditional metrics such as the citation rate and the impact factor.

Hence, researchers and publishers are exploring article-level metrics, which include not only citation rates but also potential extracted indicators such as page view, download, click, note, recommend, tag, post, trackback, and comments [14-17]. By using such multidimensional indicators, we aim to broaden researchers’ vision in the field of scientometrics and to provide richly measurable metadata for post peer review. For example, Priem and Costello [18] found Twitter citations are generated considerably more quickly than traditional citations, with 40% occurring within 1 week of the cited resource’s publication. In this paper, we call these new indicators “altmetric indicators”. Compared to traditional indicators, they are superior in terms of coverage, efficiency, and scalability.

In light of the advantage of altmetrics, many authors have called for its further evaluation. Neylon and Wu [14] noted the unsatisfactory results of traditional methods for measuring impact, and they assert that good filters of quality, importance, and relevance to apply to scientific literature are required. Taraborelli [19] suggested that collaboratively aggregated metadata may help to close the gap between traditional citation-based metrics and usage-based metrics for scientific evaluation. He also proposed that social software could be used to extract large-scale indicators of scientific quality. Priem and Hemminger [3] have likewise stated that citation-based methods poorly evaluate and filter articles and considered an examination of the usage of articles in Web 2.0 services novel and promising. They developed the most comprehensive list of Web 2.0 tools and assessed the potential value and the availability of data. Groth and Gurney [20] used keyword and citation similarity maps to analyze differences between blog posts in chemistry and in academic literature. Weller and Puschmann [21] categorized scientific tweets on Twitter and devised a method for identifying and measuring citations.

Do altmetrics correlate with traditional measures? Some researchers have studied this question and provided evidence that altmetric and traditional indicators correlate significantly. For example, Yan and Gerstein [16] examined the correlation between 18 different metrics, including article usage (HTML views, PDF downloads, XML downloads), citation statistics, blog coverage, social bookmarking, and online ratings in the PLOS Article-Level Metrics. They observed that the number of citations correlates most strongly with access statistics (*r*=.44), with the highest correlation being with number of PDF downloads (*r*=.48). Additionally, Priem et al [17] studied the correlations between 19 types of altmetric indicators and concluded that the scholarly bookmarking services Mendeley (*r*=.26) and CiteULike (*r*=.16) correlated with citations, while services such as Delicious did not. Li et al [22] investigated 1613 journal papers and studied the correlation between two online reference managers (Mendeley and CiteULike) and two types of citations (WoS and Google Scholar). Their results indicate that the Mendeley user counts significantly correlate with WoS citations, and Mendeley attracted more users than did CiteULike. Eysenbach [23] selected a cumulative number of tweetations (ie, a citation in a tweet) 7 days after article publication as tweetation counts and then calculated the correlation between citations and tweetations. The Pearson correlation coefficients for the citation versus tweetation counts were statistically significant at a 5% level and ranged from .57 to .89. Additionally, Google Scholar citations were more strongly correlated with tweetations than were Scopus citations.

In summary, all previous researchers have focused on demonstrating the performance of altmetric indicators and correlations between traditional and altmetric indicators. However, important questions such as the dimensionality and structure of altmetrics have not been explored. In other words, the overall configuration is unclear and requires further verification. For example, how many dimensions should altmetric indicators be divided into? How does the interactive structure look? Motivated by these questions, we attempt to look into the similarities and the differences between traditional and altmetric indicators. We will represent the interactive structure visually in a social network context.

For our study, it is vital to make sure altmetric indicators have the attributes of openness and maneuverability for samples before conducting an altmetrics study. The publisher platforms where articles are being written, read, and published, such as JMIR, PLOS, and social networks, such as Twitter, CiteULike, blogs, or Mendeley, where articles are being shared, recommended, discussed, and rated, make their data available through standardized application programming interfaces (APIs), which allow authors, editors, and academic administration to select the most meaningful data for a particular use at a particular time. These individuals could thus showcase a wider range of article impact in an immediate, open, and individualized way.

Article-Level Metrics represent a comprehensive set of impact indicators that capture usage, citations, social bookmarking and dissemination activity, media and blog coverage, discussion activity, and ratings. API for Article-Level Metrics is freely and publicly available. More than 150 developers have downloaded the API for data reuse to determine the total impact of articles. Hence, we consider these data to provide a good sample for our study.

On selection of the tests, we considered the options proposed by researchers, such as graduated colors for correlation coefficient matrixes [16,17] and methods that Priem used for data transformations of datasets [17]. However, we did not adopt factor analysis to disclose the clusters of altmetric indicators because the Kaiser-Meyer-Olkin (KMO) is low (KMO=0.45). Instead, we explored a nonmetric multidimensional scaling (MDS) method to reveal the dimensions of alternative metrics after nonparametric testing; presumably, there are social networks relationships between multidimensional altmetrics, so we used a social network analysis to map the altmetrics interactions.

##  Methods

We downloaded an “Article-Level Metrics” dataset (specifically, a sample of 33,128 academic articles) from the PLOS API website on December 14, 2011. The dataset includes data for a number of metrics, for example, counts of article usage, citation rates, and other types of metrics (eg, social bookmarks, comments, notes, blog posts, and ratings). We noticed that the values of the altmetric variables differ too markedly in dimensions, thus resulting in smaller absolute values weighing less when calculating the distances between values. Therefore, variables were handled as dimensionless with an algorithm “mean of 1” to keep the coefficients of the original variables constant [24].

First, we drew a histogram to discern approximately whether the data followed a normal distribution. In a normal distribution, the 2 “halves” of the histogram appear as mirror images of each other [25]. In a skewed distribution, one tail of the distribution may commonly be considerably longer or drawn out relative to the other tail. For example, in a “skewed right” distribution, the tail is on the right [26,27]. Many statistical tests are based on the assumption that the data are sampled from a normal distribution. However, when the variables are skewed (non-normal), a nonparametric test is appropriate [28]. In this paper, we also performed a one-sample Kolmogorov-Smirnov (K-S) test (a type of nonparametric test).

Second, a correlation, indicated by a correlation coefficient, measures the strength and the direction of a linear relationship between two variables [29]. For an abnormal distribution, it is more advisable to use the Spearman rank correlation than the Pearson correlation. Examining a table of coefficient numbers is impractical because a matrix of 20x20 is large, so graphical visualization tools are suitable. Various methods have been proposed, from heat maps to correlation ellipses [30]. We visualized the correlation matrix using a color graph generated by the Corrplot package in the R programming language.

Third, MDS could generate a visual representation of the subjective dimensions that are not directly indicated in the data [31]. Many applications of this method are available in bioinformatics [32,33] and ecological science [34-36]. A nonmetric MDS analysis enabled us to find a nonparametric monotonic relationship between similarities in the item-item matrix, the Euclidean distances between items, and the location of each item in the low-dimensional space [37]. We explored a nonmetric MDS analysis method with the software package UCINET to determine the types of variables that have a higher degree of similarities.

Fourth, an MDS diagram can reveal the similarities among variables, though not the strength and the structure of the relationships among variables. Visualizing the correlation matrix in a network context is useful. Researchers observe social relationships based on the theory that a social network comprises nodes and ties. Nodes represent individual actors within the network, and ties represent relationships between variables and individuals [38]. We used NetDraw (a social network analysis software package) to visualize the interaction of the variables and its strength. We also aimed to ascertain the relative importance of variables in interconnecting the network. The social network diagram helped us distinguish the number of clusters and the corresponding degrees of clustering.

## Results

### Right-Skewed Distribution and the Pareto Principle

We used a one-sample K-S test to determine whether the altmetric variables are normally distributed. In general, if *P<*.05, then the data are considered to follow an abnormal distribution [39]. Our results showed that the *P*<.001 for all variables; therefore, we rejected the normality assumption. One way to determine whether a variable is “significantly skewed” is comparing the numerical value for “skewness” with twice the standard error of skewness, including the range from minus twice to plus twice the standard error of skewness [40]. Because the skewness value falls outside this range, we concluded that the distribution is significantly non-normal and, in this case, positively skewed. Table 1 shows the integration of the results, including the K-S test, the skewness, and the kurtosis of variables. Table 2 lists the legends for B1 to B20.

**Figure 1:**
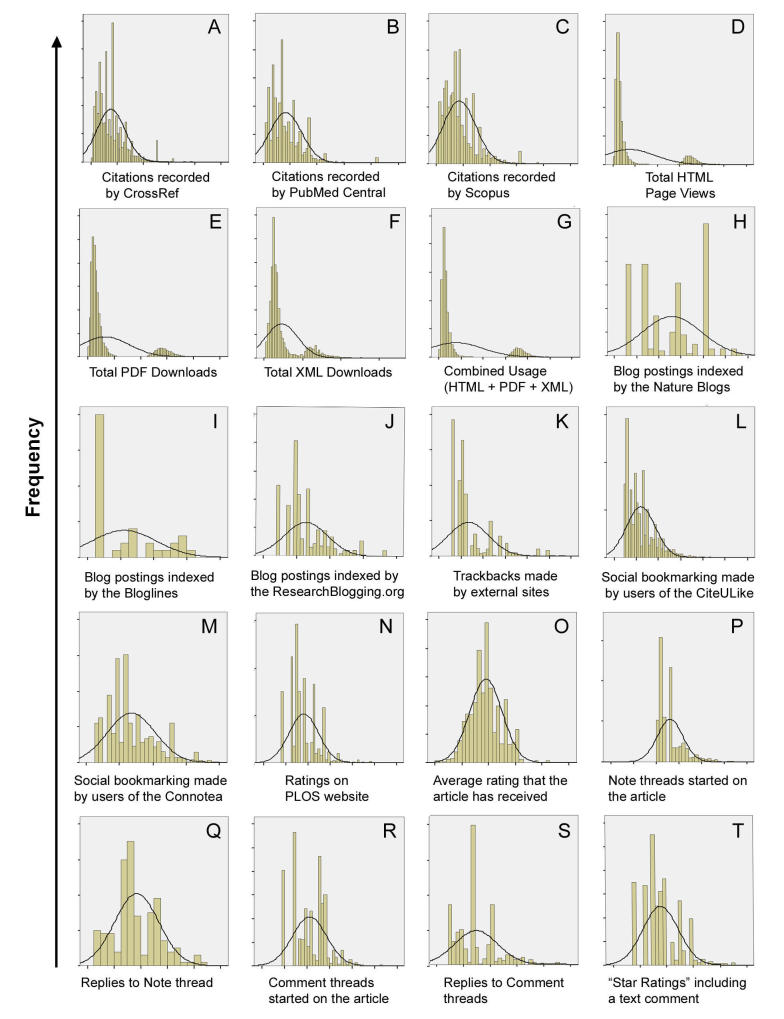
Histograms of frequency distribution for altmetric indicators.

We also drew histograms and obtained a group of skewed histograms. Because variable *B*_*i*_ is highly skewed throughout testing (the average of the skewness is 1.267, and the average of the kurtosis is 2.033), we log-transformed it into variable *D*_*i*_ (after excluding zeros) to show its distribution more clearly. Figure 1 summarizes the frequency distribution of the cumulative variable *D*_*i*_. The right tail is longer, and the distribution’s mass is concentrated on the left of the figure, thus confirming that the 20 histograms are right-skewed distributions, according to the direction of the tail. We inferred that skewness may be related to the meaning of the variables: the percentage of relative activities mentioned in the articles cannot be less than zero.

As shown in the histogram of downloads and page views (from D to G), the data have two relative peaks that follow a bimodal distribution, similar in appearance to the back of a two-humped camel. This distribution is reminiscent of the Pareto Principle (or the 80-20 rule), that is, approximately 80% of the effects arise from 20% of the causes [41,42]. With reference to the theory of knowledge scatter [43], this pattern suggested that 80% of download counts were generated by 20% of the articles.

**Table 1:** Integration of the results including K-S, skewness, and kurtosis of variables (N=33,128).

	K-S		Skewness		Kurtosis	
	Z	Asymp sig^a^ (2-tailed)	S	SE^b^	K	SE
B1	59.205	<.001	7.271	0.013	88.479	0.027
B2	73.141	<.001	15.342	0.013	299.692	0.027
B3	64.031	<.001	10.778	0.013	218.256	0.027
B4	79.442	<.001	27.596	0.013	1405.772	0.027
B5	83.492	<.001	7.902	0.013	128.074	0.027
B6	70.240	<.001	15.694	0.013	405.590	0.027
B7	81.168	<.001	21.363	0.013	908.977	0.027
B8	94.749	<.001	18.727	0.013	421.814	0.027
B9	92.407	<.001	60.796	0.013	4051.419	0.027
B10	94.019	<.001	17.715	0.013	436.739	0.027
B11	88.133	<.001	29.028	0.013	1075.095	0.027
B12	72.099	<.001	20.820	0.013	916.229	0.027
B13	93.919	<.001	16.017	0.013	359.758	0.027
B14	91.698	<.001	25.092	0.013	1010.583	0.027
B15	93.719	<.001	19.392	0.013	770.375	0.027
B16	91.612	<.001	17.786	0.013	566.946	0.027
B17	93.434	<.001	40.112	0.013	2102.985	0.027
B18	87.564	<.001	17.025	0.013	556.158	0.027
B19	92.924	<.001	32.325	0.013	1447.864	0.027
B20	94.296	<.001	27.080	0.013	1205.024	0.027

^a^asymptotic significance

^b^standard error

**Table 2:** Legends for B1 to B20.

Altmetric indicators	Legends
B1	Citations recorded by CrossRef
B2	Citations recorded by PubMed Central
B3	Citations recorded by Scopus
B4	Total HTML page views
B5	Total PDF downloads
B6	Total XML downloads
B7	Combined usage (HTML + PDF + XML)
B8	Blog postings indexed by Nature Blogs
B9	Blog postings indexed by Bloglines
B10	Blog postings indexed by ResearchBlogging.org
B11	Trackbacks made by external sites
B12	Social bookmarking made by users of CiteULike
B13	Social bookmarking made by users of Connotea
B14	Ratings on PLOS website
B15	Average rating that the article has received
B16	Note threads started on the article
B17	Replies to Note thread
B18	Comment threads started on the article
B19	Replies to Comment threads
B20	“Star Ratings” including a text comment

###  Spearman Correlation Coefficients and Their Visualization in R

We performed the normality test to conclude that neither altmetric variable is normally distributed. We used a Spearman rank order correlation to examine the correlation pattern among altmetric indicators with SPSS 18.0. Tables 3 and 4 present the results of the Spearman rank correlation coefficient for altmetric indicators.

The correlation coefficient can range from -1 to 1, with -1 or 1 indicating a perfect relationship [44]. The Spearman rho between B14 and B15 is 1. B14 represents ratings on the PLOS website, and B15 represents an article’s average rating. Therefore, it is unsurprising that the relationship is approximately perfect. The similarities in correlation strength was observed for another pair of variables (B4 and B7) with “rho=1”, likely because HTML page views (expressed by B4) accounted for the largest proportion of the combined (HTML + PDF + XML) usage of articles (expressed by B7).

The second strongest correlation, rho=.899, is between total HTML page views (expressed by B4) and total PDF downloads (expressed by B5), possibly because they are two aspects of article usage counts, and people choose view or download with approximately equivalent frequencies. The Spearman rho between B6 and B13 is –.25, so we can predict that as B6 (total XML downloads) increases, B13 (social bookmarking made by Connotea users) will decrease.

The Spearman coefficients from 91.58% of the variable pairs are significant at the .01 level, with one pair of variables (B9 and B12) correlating at the .05 significance level. Approximately no correlation exists between approximately 7.89% variable pairs. B9 (blog postings indexed by the Bloglines) also hardly correlate with eight variables (ie, B13 to B20), possibly implying that Blogline is unpopular and not widely used by researchers and citizen scientists.

The correlation matrix also yields the probability of being incorrect if we assume that the relationship observed in our sample accurately reflects the relationship among variables of altmetric indicators in the actual population from which the sample was drawn, labeled as Sig (2-tailed). We found that 91.58% of the probability value is <.001 (the value is rounded to three digits), well below the conventional threshold of *P*<.05, thus supporting our hypothesis. There is a relationship (ie, the coefficient is not 0) in the predicted direction (positive), and we can generalize the results to the population (*P*<.05).

**Figure 2:**
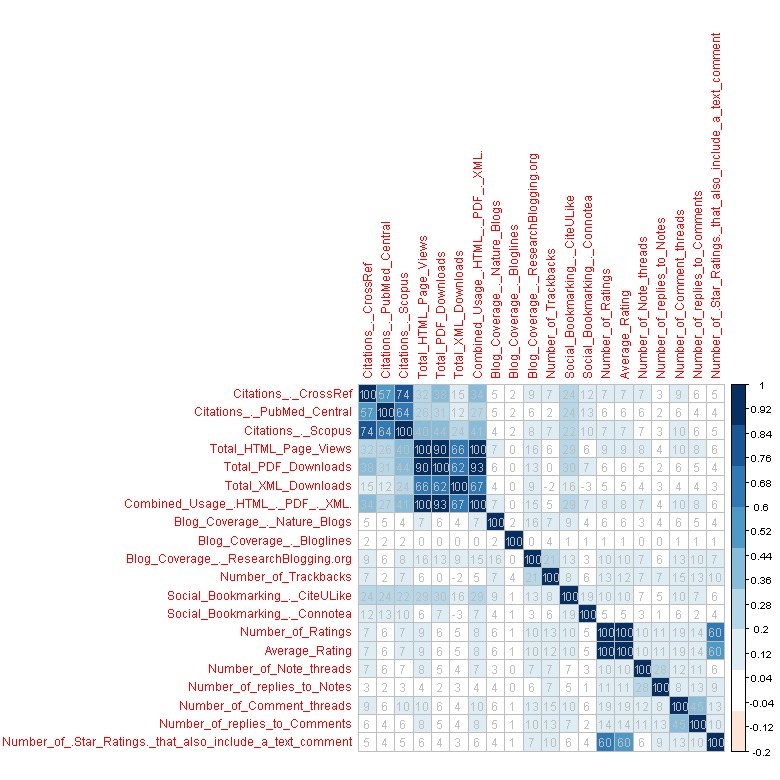
Visualization of the correlation matrix in R.

To show the correlation among altmetrics clearly, we visualized the correlation coefficient matrices with graduated colors and a blue-white-red scale. An R programming package, corrplot, helped map the correlation coefficients to the specified color square. We chose two color series to identify positive and negative correlation coefficients. Blue corresponds to a correlation of approximately 1; red to approximately –1; and white to approximately 0. To economize space, we multiplied the correlation coefficients by 100 and added them to the squares in the color correlation matrix. See Figure 2.

We can readily identify clusters with strong similarities and locate possible redundant indicators. Matching this map with the physical meaning revealed the following: (1) the citation indicators (B1, B2, and B3) and download indicators (B4, B5, B6, and B7) are clustered into two categories, which we call the “citation metrics class” and “download metrics class”, respectively; (2) the citation and download indicators are combined into a clustering, which we call the “traditional metrics class”; (3) a group of indicators (B14 to B20) are conjoined into another clustering type, called the “rating, note, and comment metrics class”; and (4) finally, as a general rule, we suggested that all four blog-aggregating services would record different sets of data, so the datasets require comparison and “de-duplication” to obtain a complete picture of blog activity (as recorded by these services), as would all three citation services.

**Table 3:** Spearman rank correlation coefficient for B1-B11 (N=33,128).

		B1	B2	B3	B4	B5	B6	B7	B8	B9	B10
B1	Corr. coefficient	1.000	.599^a^	.738^a^	.322^a^	.378^a^	.153^a^	.338^a^	.050^a^	.019^a^	.088^a^
	Sig (2-tailed)		<.001	<.001	<.001	<.001	<.001	<.001	<.001	<.001	<.001
B2	Corr. coefficient	.599^a^	1.000	.669^a^	.226^a^	.268^a^	.079^a^	.237^a^	.051^a^	.023^a^	.063^a^
	Sig (2-tailed)	<.001		<.001	<.001	<.001	<.001	<.001	<.001	<.001	<.001
B3	Corr. coefficient	.738^a^	.669^a^	1.000	.402^a^	.444^a^	.244^a^	.415^a^	.040^a^	.020^a^	.084^a^
	Sig (2-tailed)	<.001	<.001		<.001	<.001	<.001	<.001	<.001	<.001	<.001
B4	Corr. coefficient	.322^a^	.226^a^	.402^a^	1.000	.899^a^	.662^a^	.996^a^	.070^a^	-.002	.156^a^
	Sig (2-tailed)	<.001	<.001	<.001		<.001	<.001	<.001	<.001	.784	<.001
B5	Corr. coefficient	.378^a^	.268^a^	.444^a^	.899^a^	1.000	.616^a^	.928^a^	.065^a^	.002	.125^a^
	Sig (2-tailed)	<.001	<.001	<.001	<.001		<.001	<.001	<.001	.695	<.001
B6	Corr. coefficient	.153^a^	.079^a^	.244^a^	.662^a^	.616^a^	1.000	.672^a^	.039^a^	-.005	.089^a^
	Sig (2-tailed)	<.001	<.001	<.001	<.001	<.001		<.001	<.001	.399	<.001
B7	Corr. coefficient	.338^a^	.237^a^	.415^a^	.996^a^	.928^a^	.672^a^	1.000	.070^a^	-.001	.153^a^
	Sig (2-tailed)	<.001	<.001	<.001	<.001	<.001	<.001		<.001	.892	<.001
B8	Corr. coefficient	.050^a^	.051^a^	.040^a^	.070^a^	.065^a^	.039^a^	.070^a^	1.000	.019^a^	.158^a^
	Sig (2-tailed)	<.001	<.001	<.001	<.001	<.001	<.001	<.001		<.001	<.001
B9	Corr. coefficient	.019^a^	.023^a^	.020^a^	-.002	.002	-.005	-.001	.019^a^	1.000	-.001
	Sig (2-tailed)	<.001	<.001	<.001	.784	.695	.399	.892	<.001		.918
B10	Corr. coefficient	.088^a^	.063^a^	.084^a^	.156^a^	.125^a^	.089^a^	.153^a^	.158^a^	-.001	1.000
	Sig (2-tailed)	<.001	<.001	<.001	<.001	<.001	<.001	<.001	<.001	.918	
B11	Corr. coefficient	.072^a^	.061^a^	.073^a^	.063^a^	-.004	-.015^a^	.053^a^	.071^a^	.035^a^	.214^a^
	Sig (2-tailed)	<.001	<.001	<.001	<.001	.426	.006	<.001	<.001	<.001	<.001
B12	Corr. coefficient	.240^a^	.248^a^	.222^a^	.288^a^	.299^a^	.156^a^	.293^a^	.086^a^	.014^b^	.128^a^
	Sig (2-tailed)	<.001	<.001	<.001	<.001	<.001	<.001	<.001	<.001	.014	<.001
B13	Corr. coefficient	.120^a^	.159^a^	.102^a^	.065^a^	.071^a^	-.025^a^	.067^a^	.042^a^	.010	.031^a^
	Sig (2-tailed)	<.001	<.001	<.001	<.001	<.001	<.001	<.001	<.001	.071	<.001
B14	Corr. coefficient	.074^a^	.075^a^	.072^a^	.087^a^	.057^a^	.050^a^	.085^a^	.055^a^	.005	.101^a^
	Sig (2-tailed)	<.001	<.001	<.001	<.001	<.001	<.001	<.001	<.001	.328	<.001
B15	Corr. coefficient	.074^a^	.076^a^	.072^a^	.087^a^	.057^a^	.050^a^	.085^a^	.055^a^	.005	.101^a^
	Sig (2-tailed)	<.001	<.001	<.001	<.001	<.001	<.001	<.001	<.001	.350	<.001
B16	Corr. coefficient	.069^a^	.061^a^	.067^a^	.075^a^	.053^a^	.042^a^	.073^a^	.028^a^	-.001	.070^a^
	Sig (2-tailed)	<.001	<.001	<.001	<.001	<.001	<.001	<.001	<.001	.794	<.001
B17	Corr. coefficient	.027^a^	.021^a^	.027^a^	.045^a^	.024^a^	.026^a^	.043^a^	.036^a^	-.002	.058^a^
	Sig (2-tailed)	<.001	<.001	<.001	<.001	<.001	<.001	<.001	<.001	.662	<.001
B18	Corr. coefficient	.090^a^	.101^a^	.096^a^	.099^a^	.056^a^	.043^a^	.097^a^	.063^a^	.010	.133^a^
	Sig (2-tailed)	<.001	<.001	<.001	<.001	<.001	<.001	<.001	<.001	.063	<.001
B19	Corr. coefficient	.058^a^	.055^a^	.057^a^	.082^a^	.053^a^	.041^a^	.079^a^	.052^a^	.008	.103^a^
	Sig (2-tailed)	<.001	<.001	<.001	<.001	<.001	<.001	<.001	<.001	.137	<.001
B20	Corr. coefficient	.050^a^	.049^a^	.049^a^	.062^a^	.043^a^	.035^a^	.060^a^	.037^a^	.009	.073^a^
	Sig (2-tailed)	<.001	<.001	<.001	<.001	<.001	<.001	<.001	<.001	.090	<.001

^a^Correlation is significant at the .01 level (2-tailed).

^b^Correlation is significant at the .05 level (2-tailed).

**Table 4:** Spearman rank correlation coefficient for B12-B20 (N=33,128).

		B11	B12	B13	B14	B15	B16	B17	B18	B19	B20
B1	Corr. coefficient	.072^a^	.240^a^	.120^a^	.074^a^	.074^a^	.069^a^	.027^a^	.090^a^	.058^a^	.050^a^
	Sig (2-tailed)	<.001	<.001	<.001	<.001	<.001	<.001	<.001	<.001	<.001	<.001
B2	Corr. coefficient	.061^a^	.248^a^	.159^a^	.075^a^	.076^a^	.061^a^	.021^a^	.101^a^	.055^a^	.049^a^
	Sig (2-tailed)	<.001	<.001	<.001	<.001	<.001	<.001	<.001	<.001	<.001	<.001
B3	Corr. coefficient	.073^a^	.222^a^	.102^a^	.072^a^	.072^a^	.067^a^	.027^a^	.096^a^	.057^a^	.049^a^
	Sig (2-tailed)	<.001	<.001	<.001	<.001	<.001	<.001	<.001	<.001	<.001	<.001
B4	Corr. coefficient	.063^a^	.288^a^	.065^a^	.087^a^	.087^a^	.075^a^	.045^a^	.099^a^	.082^a^	.062^a^
	Sig (2-tailed)	<.001	<.001	<.001	<.001	<.001	<.001	<.001	<.001	<.001	<.001
B5	Corr. coefficient	-.004	.299^a^	.071^a^	.057^a^	.057^a^	.053^a^	.024^a^	.056^a^	.053^a^	.043^a^
	Sig (2-tailed)	.426	<.001	<.001	<.001	<.001	<.001	<.001	<.001	<.001	<.001
B6	Corr. coefficient	-.015^a^	.156^a^	-.025^a^	.050^a^	.050^a^	.042^a^	.026^a^	.043^a^	.041^a^	.035^a^
	Sig (2-tailed)	.006	<.001	<.001	<.001	<.001	<.001	<.001	<.001	<.001	<.001
B7	Corr. coefficient	.053^a^	.293^a^	.067^a^	.085^a^	.085^a^	.073^a^	.043^a^	.097^a^	.079^a^	.060^a^
	Sig (2-tailed)	<.001	<.001	<.001	<.001	<.001	<.001	<.001	<.001	<.001	<.001
B8	Corr. coefficient	.071^a^	.086^a^	.042^a^	.055^a^	.055^a^	.028^a^	.036^a^	.063^a^	.052^a^	.037^a^
	Sig (2-tailed)	<.001	<.001	<.001	<.001	<.001	<.001	<.001	<.001	<.001	<.001
B9	Corr. coefficient	.035^a^	.014^b^	.010	.005	.005	-.001	-.002	.010	.008	.009
	Sig (2-tailed)	<.001	.014	.071	.328	.350	.794	.662	.063	.137	.090
B10	Corr. coefficient	.214^a^	.128^a^	.031^a^	.101^a^	.101^a^	.070^a^	.058^a^	.133^a^	.103^a^	.073^a^
	Sig (2-tailed)	<.001	<.001	<.001	<.001	<.001	<.001	<.001	<.001	<.001	<.001
B11	Corr. coefficient	1.000	.078^a^	.059^a^	.125^a^	.124^a^	.073^a^	.068^a^	.148^a^	.128^a^	.096^a^
	Sig (2-tailed)		<.001	<.001	<.001	<.001	<.001	<.001	<.001	<.001	<.001
B12	Corr. coefficient	.078^a^	1.000	.194^a^	.098^a^	.097^a^	.067^a^	.045^a^	.097^a^	.073^a^	.064^a^
	Sig (2-tailed)	<.001		<.001	<.001	<.001	<.001	<.001	<.001	<.001	<.001
B13	Corr. coefficient	.059^a^	.194^a^	1.000	.050^a^	.049^a^	.031^a^	.007	.060^a^	.023^a^	.039^a^
	Sig (2-tailed)	<.001	<.001		<.001	<.001	<.001	.215	<.001	<.001	<.001
B14	Corr. coefficient	.125^a^	.098^a^	.050^a^	1.000	1.000^a^	.097^a^	.109^a^	.190^a^	.143^a^	.602^a^
	Sig (2-tailed)	<.001	<.001	<.001		<.001	<.001	<.001	<.001	<.001	<.001
B15	Corr. coefficient	.124^a^	.097^a^	.049^a^	1.000^a^	1.000	.096^a^	.107^a^	.189^a^	.141^a^	.599^a^
	Sig (2-tailed)	<.001	<.001	<.001	<.001		<.001	<.001	<.001	<.001	<.001
B16	Corr. coefficient	.073^a^	.067^a^	.031^a^	.097^a^	.096^a^	1.000	.283^a^	.116^a^	.112^a^	.065^a^
	Sig (2-tailed)	<.001	<.001	<.001	<.001	<.001		<.001	<.001	<.001	<.001
B17	Corr. coefficient	.068^a^	.045^a^	.007	.109^a^	.107^a^	.283^a^	1.000	.085^a^	.132^a^	.094^a^
	Sig (2-tailed)	<.001	<.001	.215	<.001	<.001	<.001		<.001	<.001	<.001
B18	Corr. coefficient	.148^a^	.097^a^	.060^a^	.190^a^	.189^a^	.116^a^	.085^a^	1.000	.448^a^	.127^a^
	Sig (2-tailed)	<.001	<.001	<.001	<.001	<.001	<.001	<.001		<.001	<.001
B19	Corr. coefficient	.128^a^	.073^a^	.023^a^	.143^a^	.141^a^	.112^a^	.132^a^	.448^a^	1.000	.105^a^
	Sig (2-tailed)	<.001	<.001	<.001	<.001	<.001	<.001	<.001	<.001		<.001
B20	Corr. coefficient	.096^a^	.064^a^	.039^a^	.602^a^	.599^a^	.065^a^	.094^a^	.127^a^	.105^a^	1.000
	Sig (2-tailed)	<.001	<.001	<.001	<.001	<.001	<.001	<.001	<.001	<.001	

^a^Correlation is significant at the .01 level (2-tailed).

^b^Correlation is significant at the .05 level (2-tailed).

### Nonmetric MDS With UCINET and Network Visualization With NetDraw

**Figure 3:**
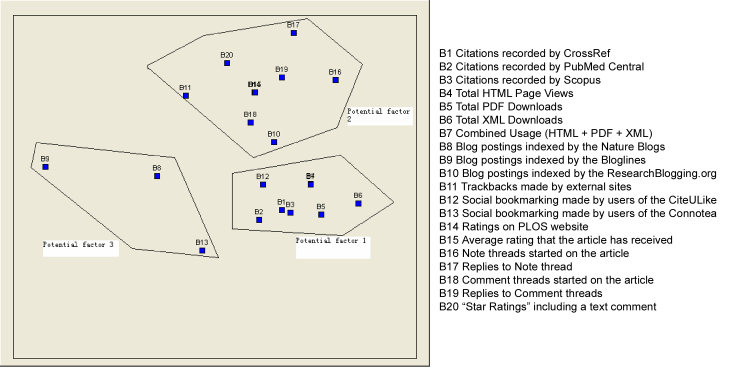
MDS diagram of altmetric indicators.

Nonmetric MDS is often preferred because it tends to provide a better “goodness-of-fit” (stress) statistic, which is correspondingly better with lower stress (0=perfect fit) [45]. Generally, stress levels below 0.1 are considered excellent, while levels above 0.2 are considered unacceptable. Accordingly, a higher RSQ (r-squared) value (1=perfect fit) is better, and RSQ values exceeding 0.6 are usually considered excellent [46]. We conducted nonmetric MDS with UCINET 6. The output map is shown in Figure 3.

The reliability value stress was 0.00424, considerably less than 0.1, and the validity value RSQ was 0.99998, greater than 0.60, which equals an excellent goodness of fit. The map plots each variable, thus permitting us to examine the similarity according to the variables’ proximity to each other. We labeled three dimensions, or categories, with each dimension implicating a potential factor.

The three clusters and their interpretations are as follows. (1) The first cluster contains B1 to B7 and B12. This cluster has 8 spots, and they are more interconnected. B4 and B7 occupy approximately the same coordinate. This cluster implicates a potential factor of 1, which we call a traditional metrics group because 7 out of 8 indicators in this cluster are citation and download indicators. (2) The second cluster contains B10, B11, and B14 to B20. This cluster has 9 spots, and they are more interconnected. B14 and B15 occupy approximately the same coordinate. This cluster implicates a potential factor of 2, and we call it the trackback, rating, note, and comment metrics group. (3) The third cluster contains B8, B9, and B13. This cluster has 3 spots, yet they are less interconnected, with more diverse networks. This cluster implicates a potential factor of 3, and we call it the blog and social bookmark metrics group.

**Figure 4:**
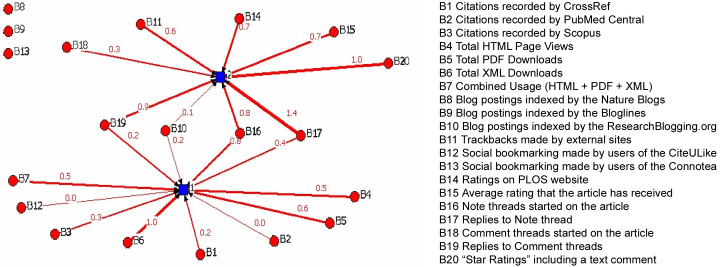
Social network structure diagram of altmetric indicators.

We know that an MDS graph can represent the relations among nodes, while a network diagram can describe the social structure. Hence, we visualized the results of the nonmetric MDS from a network context with NetDraw (version 2.084, which is distributed with UCINET 6). The network diagram is shown in Figure 4.

A good drawing of a graph can immediately suggest some of the most important features of the overall network structure. The diagram indicates the following findings: (1) not all nodes are connected, as three nodes (B8, B9, and B13) that are disconnected from the others; (2) two subgroups or local “clusters” of actors are tied to each other, not to other groups, and (3) some actors have many ties, and some, few ties. Four nodes (B10, B16, B17, and B19) have two ties, while the other nodes have one tie or zero ties. These nodes are embedded in the neighborhood by the two clusters; that is, they are important for connecting the two clusters, which we call cluster 1 and cluster 2. Thus, examining the node and the “node network” (ie, “neighborhood”) indicates a sense of the structural constraints and opportunities that an actor faces and may help us to understand an actor’s role in a social structure. Finally, it indicates that (4) some difference in the strength of the relationship between a multivariable and its center remain. For example, B12 and B2 have a weak relationship with their center, while B6 and B20 have a relatively stronger relationship (that is, “1.0”) with their centers, and B17 has the strongest relationship (that is, “1.4”) with its center.

## Discussion

### Principal Findings

Our study is the first to use the MDS and network map to analyze the dimensions and interactions among altmetrics variables. Although MDS diagrams have been used for co-citation [47] and co-word analysis [48], it is still innovative to draw nonmetric MDS diagrams for altmetrics variables. We found three dimensions or metrics groups, that is, traditional metrics (citation and download metrics), active altmetrics (trackback, rating, note, and comment metrics), and inactive altmetrics (blog and social bookmark metrics).

More importantly, we transformed the MDS diagram into a social network graph, whose advantage is that it displays the overall network structure. In research related to altmetrics, authors have developed co-word social network maps for articles published in blogs [20]. Our map represents the MDS diagram in a social network context. We found that the ResearchBlogging.org posts, note threads, and replies to comment threads are the three intermediary metrics between traditional metrics and active altmetrics; in other words, they possess attributes of traditional metrics and active altmetrics.

What do these findings imply? There may be a transfer phenomenon for social impact of academic articles. Then, Figure 4 could be considered an article impact transitive map. Along the impact transfer path, B10, B16, B17, and B19 are the transfer, or intermediate, stations that transport article social impact between active altmetrics and traditional metrics. Aman [49] quantified the extent to which preprints in arXiv accelerate scholarly communication using many subject samples. He found that, in all fields except biology, a significant citation advantage exists in terms of speed and citation rates for articles with a previous preprint version on arXiv. Shuai et al [50] studied whether Wikipedia shapes academic impact and showed that articles mentioned on Wikipedia have higher citations than do unmentioned articles. Our finding of altmetrics interactions posits that an intermediate station and a potential pathway may exist by which impact activator arXiv, Wikipedia, or other open access platforms and social network tools likely help articles attract more online usage, in turn accelerating online social activities such as comment, note, post, rate, or bookmark and thus expanding an article’s social influence, reflected in larger citation rates and higher dissemination speed. This results in the observation that altmetrics is the superior way to look at publications.

Another finding is that altmetrics correlate with traditional measures significantly; that the citation and download metrics cluster closely together by the Spearman correlation method is consistent with previous results [17] to some extent. This is exemplified by the correlation between citation counts and access statistics (*r*=.30); the highest correlation being with number of PDF downloads (*r*=.44); and the correlations between citations and scholarly bookmarking services CiteULike (*r*=.24) and Connotea (*r*=.13). Before studying the correlation of altmetric indicators, we looked more closely at the choice of method for skewed data. However, the Spearman and color square visualization methods we used differ from the methods used in previous research. For example, the Pearson, not the Spearman correlation method, was used by Eysenbach [23], while our study added a color square visualization method to better reveal correlations. Furthermore, Yan [16] found that article access metrics, citation metrics, and social bookmarking metrics broadly cluster, a formation signified by relatively high correlation coefficients among the metrics; we found additional clusters such as the “rating, note, and comment metrics class”. Moreover, we came up with a “traditional metrics class”, which integrates the “citation metrics class” and the “download metrics class”.

Our third contribution is the adoption of the theory of nonparametric testing throughout analysis. Based on the one-sample K-S test and the shapes of the histograms, we concluded that the distribution is significantly non-normal and positively skewed. Priem summarized a group of histograms similarly but did not perform a nonparametric test to prove the abnormal distribution or to compute the skewness [17]. We calculated the Spearman coefficients (obtained by nonparametric measures), not Pearson coefficients, to calculate the correlation strengths. Although the Spearman measure has been used by Yan and Gerstein [16], their sample size (13,000 articles) was smaller than ours (33,128 articles). More importantly, the Spearman coefficient is statistically fit for abnormal datasets. Additionally, the nonmetric MDS employed to detect the similarity of the variables is also a nonparametric test.

Our results also support that altmetric indicators may obey certain rules, for example, the Pareto law. Eysenbach [23] was the first to report the Pareto law for tweetation (one of the altmetric indicators). He mined all tweets containing links to articles in the *Journal of Medical Internet Research* between July 2008 and November 2011. He explored the dynamics, the content, and the timing of tweets based on a subset of 1573 tweets on approximately 55 articles and found an uneven distribution in which the top 20% of the tweet authors, as ranked by number of tweetations, accounted for 63.4% of all tweetations. This tweetation regularity follows a Pareto distribution (80/20 rule). Similarly, our frequency distribution histograms from D to G (four types of altmetric indicators) indicate that the top 20% of articles triggered 80% of download and page views and thus verified that the distribution follows the Pareto law. Therefore, we offer a complementary explanation of the Pareto regularities using altmetric indicators.

Based on our experimental results, we conclude that altmetrics complements traditional statistics and contains approximately three dimensions: traditional, active, and inactive metrics. In summary, our study demonstrates a novel interaction among the altmetrics variables and analyzes articles’ social impact transfer mechanism.

Our conclusion that the distribution is significantly non-normal and positively skewed rests primarily on the results obtained with the Article-Level Metrics dataset downloaded from PLOS API. Both Priem [17] and Yan [16] studied altmetrics based on a similar dataset. Our views regarding whether the distribution of variables is normal are consistent with theirs. However, we demonstrated the necessity of nonparameter testing in analyzing the altmetrics dataset.

### Limitations

However, as alternative metrics indicators are preset in the dataset, the implication of our study’s findings is limited. This study was a preliminary attempt, and we are preparing to test and verify these findings for other types of datasets. The findings of correlations have been confirmed by another dataset concerning altmetric indicators in [22] and [23]. Further research is required on the dimension, structure, and potential impact transfer mechanism.

### Conclusions

In conclusion, we studied the dimension and the structure of altmetrics with visual graphics. Our findings provide an important direction regarding the current practices of authors, editors, and academic administrations. Authors should pay more attention to the scholarly social impact that originates from active altmetrics and then participate more in related activities such as rating websites, noting, and commenting on articles. The publishers should attempt to launch an open peer review and consider scientific citizens’ perspectives before deciding whether to publish. They should also explore the value and the applications of post-publication interactivity in terms of ratings, notes, or comments. Academic administrations should track the dissemination of published articles (in terms of multiple types of citation, ratings, comments, and notes) and access up-to-date altmetrics data to determine article quality or the impact context for tenure and promotion decisions.

## Abbreviations

API: application programming interface
KMO: Kaiser-Meyer-Olkin
K-S tests: Kolmogorov-Smirnov Test
MDS: multidimensional scale
RSQ: r-squared

## References

[1] Abbott A, Cyranoski D, Jones N, Maher B, Schiermeier Q, Van Noorden R (2010). Metrics: Do metrics matter?. Nature.

[2] Alex G, Holmberg Kim, Christina K, Heather P, Jason P, Nicholas w (2011). Embracing new methods for publishing, finding, discussing, and measuring our research output. Proceedings of the American Society for Information Science and Technology.

[3] Jason P, Bradely H (2010). First Monday.

[4] Paul S, Nicholas C (2011). How to improve your impact factor: Questioning the quantification of academic quality. Journal of Philosophy of Education.

[5] Reinstein A, Hasselback JR, Riley ME, Sinason DH (2011). Pitfalls of using citation indices for making academic accounting promotion, tenure, teaching load, and merit pay decisions. Issues in Accounting Education.

[6] Ramalho Correia AM, Teixeria JC (2005). Reforming scholarly publishing and knowledge communication: From the advent of the scholarly journal to the challenges of open access. Online Information Review.

[7] Willinsky J (2003). The nine flavours of open access scholarly publishing. J Postgrad Med.

[8] Greenhow C (2009). Knowledge Quest.

[9] Veletsianos G, Kimmons R (2012). Networked Participatory Scholarship: Emergent techno-cultural pressures toward open and digital scholarship in online networks. Computers & Education.

[10] Ebner M, Reinhardt W (2009). Social networking in scientiﬁc conferences –Twitter as tool for strengthening a scientiﬁc community. Proceedings of the 1st International Workshop on Science.

[11] Kjellberg S (2010). First Monday.

[12] Kirkup G (2010). Academic blogging: academic practice and academic identity. London Review of Education.

[13] Wang ML (2011). The impact of open access journals on library and information scientists' research in Taiwan. http://eprints.uitm.edu.my/3624/1/SP_TIO11_61.pdf.

[14] Neylon C, Wu S (2009). Article-level metrics and the evolution of scientific impact. PLoS Biol.

[15] Bollen J, Van de Sompel H, Hagberg A, Bettencourt L, Chute R, Rodriguez MA, Balakireva L (2009). Clickstream data yields high-resolution maps of science. PLoS One.

[16] Yan KK, Gerstein M (2011). The spread of scientific information: insights from the web usage statistics in PLoS article-level metrics. PLoS One.

[17] Jason P, Heather A, Bradley H (2011). Altmetrics in the mild: An exploratory study of impact metrics based on social media. Metrics 2011.

[18] Priem J, Costello KL (2011). How and why scholars cite on Twitter. Proc Am Soc Info Sci Tech.

[19] Taraborelli D (2008). Soft peer review: social software and distributed scientific evaluation. http://eprints.ucl.ac.uk/8279/.

[20] Groth P, Gurney T (2010). Studying scientific discourse on the web using bibliometrics: a chemistry blogging case study. Proceedings of the WebSci10: Extending the Frontiers of Society On-Line.

[21] Weller K, Puschmann C (2011). Twitter for scientific communication: How can citations/references be identified and measured. http://journal.webscience.org/500/1/153_paper.pdf.

[22] Li X, Thelwall M, Giustini D (2011). Validating online reference managers for scholarly impact measurement. Scientometrics.

[23] Eysenbach G (2011). Can tweets predict citations? Metrics of social impact based on Twitter and correlation with traditional metrics of scientific impact. J Med Internet Res.

[24] Vidakovic B (2011). Chapter 6: Normal distribution. Statistics for Bioengineering Sciences: With MATLAB and WinBUGS Support.

[25] Carroll C, Paul T, James J, Barry H, William G, Ledi T, Jack P (2003). NIST/SEMATECH e-Handbook of Statistical Methods.

[26] Howitt D, Cramer D (2010). Shapes of distributions of scores. Introduction to Statistics in Psychology.

[27] Babak S, Robert G, Kurt H, Giovanni P (2012). Exploring numerical variables. Biostatistics with R: An introduction to statistics through biological data.

[28] Motulsky H (2010). The Gaussian distribution. Intuitive biostatistics: a nonmathematical guide to statistical thinking.

[29] Yeh S-T, GlaxoSmithKline, King of Prussia, PA (2007). Exploratory visualization of correlation matrices. http://www.nesug.org/proceedings/nesug07/np/np18.pdf.

[30] David S (2009). Revolutions: a blog dedicated to news and information of interest to members of the R community.

[31] Bronstein AM, Bronstein MM, Kimmel R (2006). Generalized multidimensional scaling: a framework for isometry-invariant partial surface matching. Proceedings of the National Academy of Sciences of the United States of America (PNAS).

[32] Antoine N, Wlodzislaw D (2000). Interactive data exploration using Mds mapping (2000). http://citeseerx.ist.psu.edu/viewdoc/download;jsessionid=6425975A7E49D901E17A3A1E94F418FF?doi=10.1.1.35.2761&rep=rep1&type=pdf.

[33] Taguchi YH, Oono Y (2005). Relational patterns of gene expression via non-metric multidimensional scaling analysis. Bioinformatics.

[34] Kenkel N, Orloci L (1986). Applying metric and nonmetric multidimensional scaling to ecological studies: Some new results. Ecology.

[35] János P (2005). Multivariate exploratory analysis of ordinal data in ecology: Pitfalls, problems and solutions. Journal of Vegetation Science.

[36] Holger K, Walter J (2010). A framework for delineating biogeographical regions based on species distributions. Journal of Biogeography.

[37] Wikipedia.

[38] Wasserman S, Katherine F (1994). Social Network Analysis: Methods and Applications (Structural Analysis in the Social Sciences.

[39] Paul A, Venita D (2004). An Overview of Non-parametric Tests in SAS: When, Why, and How.

[40] (2000). Determining if skewness and kurtosis are significantly non-normal.

[41] Beth E, Cristina M, Randolph G, Donald G (2006). Behavioral citation analysis: Toward collection enhancement for users. College & Research Libraries.

[42] Wilson CS, Tenopir C (2008). Local citation analysis, publishing and reading patterns: Using multiple methods to evaluate faculty use of an academic library's research collection. J Am Soc Inf Sci.

[43] Tonta Y, Al U (2006). Scatter and obsolescence of journals cited in dissertations of librarianship. Library & Information Science Research.

[44] Edward N, Nan C, Hecht L, Nelson E, Ross J, Fiddler L (2000). Correlation and regression. SPSS for Windows Version 11.0: A Basic Tutorial.

[45] Sean F (2004). A guide for the visually perplexed: visually representing social networks.

[46] Borgatti SP, Everett MG, Freeman LC (2002). Ucinet for Windows: Software for Social Network Analysis.

[47] Zhao DZ (2006). Towards all-author co-citation analysis. Information Processing and Management: an International Journal - Special issue: Informetrics.

[48] Vaughan L, Yang RB, Chen C, Liang WB, Li BY (2010). Extending web co-link analysis to web co-word analysis for competitive intelligence. Proceedings of the Annual Conference of the Canadian Association for Information Science.

[49] Valeria A (2013). arXiv.

[50] Shuai X, Jiang ZR, Liu XZ, Johan B (2013). A comparative study of academic impact and Wikipedia ranking. Proceedings of the 13th ACM/IEEE-CS joint conference on Digital libraries.

